# Supplementary material for: Mechanisms governing the pioneering and redistribution capabilities of the non-classical pioneer PU.1
Source: Nat Commun. 2020 Jan 21;11:402. doi: 10.1038/s41467-019-13960-2 (PMC6972792; doi:10.1038/s41467-019-13960-2)
Supplement: Supplementary file 7 — Source data [file 41467_2019_13960_MOESM7_ESM.zip › Source_Data/Figure6/Figure6B_MotifScanOutput/FLI1_PU.1/homerResults/motif1.similar.html]

motif1

## Information for motif1

C
T
G
A
A
G
T
C
G
C
A
T
A
C
G
T
A
G
T
C
A
G
T
C
A
C
G
T
A
T
C
G
G
C
A
T
A
G
C
T
  
Reverse Opposite:  

T
C
G
A
C
G
T
A
T
A
G
C
T
G
C
A
C
T
A
G
A
C
T
G
C
G
T
A
C
G
T
A
T
C
A
G
G
A
C
T
  

|  |  |
| --- | --- |
| p-value: | 1e-2892 |
| log p-value: | -6.661e+03 |
| Information Content per bp: | 1.616 |
| Number of Target Sequences with motif | 8182.0 |
| Percentage of Target Sequences with motif | 82.24% |
| Number of Background Sequences with motif | 7282.4 |
| Percentage of Background Sequences with motif | 19.96% |
| Average Position of motif in Targets | 101.6 +/- 43.8bp |
| Average Position of motif in Background | 99.0 +/- 74.7bp |
| Strand Bias (log2 ratio + to - strand density) | -0.0 |
| Multiplicity (# of sites on avg that occur together) | 1.66 |
| Motif File: | file (matrix) reverse opposite |

### Similar de novo motifs found

|  |  |  |  |  |  |  |  |
| --- | --- | --- | --- | --- | --- | --- | --- |
| Rank | Match Score | Redundant Motif | P-value | log P-value | % of Targets | % of Background | Motif file |
| 1 | 0.959 | G T C A T A G C A G T C G A T C T C G A A G T C G C A T A C G T A G T C G A T C A C G T A T C G G A C T A G C T | 1e-2837 | -6533.920537 | 84.72% | 22.76% | motif file (matrix) |
| 2 | 0.963 | T C G A A G T C C G A T A C G T A G T C A G T C A C T G A C T G G A T C | 1e-2811 | -6473.363764 | 86.49% | 24.78% | motif file (matrix) |
| 3 | 0.968 | A T G C A G T C G A T C T C G A A G T C G C A T A C G T A G T C A G T C A C G T A T C G G A C T A G C T | 1e-2772 | -6384.015790 | 84.96% | 23.63% | motif file (matrix) |
| 4 | 0.959 | C G T A T G A C G T C A C T A G C T A G C G T A C G T A T C A G G A C T A T C G T G A C T A G C | 1e-2676 | -6162.023315 | 80.51% | 20.47% | motif file (matrix) |
| 5 | 0.892 | G T A C A C T G A C T G C G T A C G T A C T G A A G T C | 1e-2486 | -5726.409421 | 83.97% | 25.62% | motif file (matrix) |
| 6 | 0.932 | T A C G T G A C T C A G T C A G G C T A C G T A T C A G A G C T C T A G C T A G | 1e-2443 | -5626.684783 | 92.47% | 36.18% | motif file (matrix) |
| 7 | 0.973 | C T G A C T G A T A G C T G C A C T A G A C T G C G T A C G T A T C A G G A C T T C G A | 1e-2381 | -5483.190425 | 83.01% | 25.83% | motif file (matrix) |
| 8 | 0.948 | T A G C T G C A C T A G A C T G C G T A C T G A C T A G G A C T | 1e-1976 | -4550.937120 | 83.13% | 30.80% | motif file (matrix) |
| 9 | 0.910 | T G A C T C A G A T C G G C T A G C T A T C A G G A C T T C A G | 1e-1939 | -4465.839563 | 72.48% | 21.25% | motif file (matrix) |
| 10 | 0.916 | G A T C A G C T C G T A A T G C A C G T A C G T A G T C A G T C A C G T A T C G C G T A C G A T T C G A | 1e-1796 | -4136.190571 | 91.02% | 43.12% | motif file (matrix) |
| 11 | 0.912 | C G A T C G A T G T C A T G A C G T C A A C T G A C T G C G T A C G T A T C G A T C G A C T A G T A G C C A G T | 1e-1694 | -3901.619077 | 96.97% | 54.92% | motif file (matrix) |
| 12 | 0.886 | C G A T A C G T A G T C A G T C A C T G A T C G G A C T | 1e-1364 | -3140.891599 | 48.01% | 10.42% | motif file (matrix) |
| 13 | 0.936 | C T G A C G T A T A C G T G C A C T A G A C T G G T C A C G T A T A C G | 1e-1362 | -3137.690786 | 59.32% | 17.79% | motif file (matrix) |
| 14 | 0.910 | C G A T C G A T T A G C A G T C A C T G A T G C G A C T A G C T | 1e-1265 | -2913.743804 | 69.51% | 27.41% | motif file (matrix) |
| 15 | 0.917 | T C G A A G T C C G A T C G A T A G T C A G T C A C T G G T A C G A C T | 1e-1208 | -2783.014082 | 70.99% | 29.65% | motif file (matrix) |
| 16 | 0.888 | T C G A C T G A T A C G T C G A C T A G T C A G T C G A C T G A T C A G G C A T C T G A T C A G | 1e-1193 | -2747.496469 | 75.12% | 33.94% | motif file (matrix) |
| 17 | 0.890 | C T A G T C G A C T A G A C T G C G T A C G T A T C A G A G C T C A T G | 1e-1132 | -2606.651461 | 62.09% | 23.03% | motif file (matrix) |
| 18 | 0.815 | A G C T A T G C G C T A A T G C C G A T C G A T T A G C A G T C C A T G | 1e-1092 | -2514.588877 | 42.75% | 10.09% | motif file (matrix) |
| 19 | 0.839 | A T G C A C G T A C G T A G T C A G T C A G C T A G T C | 1e-1018 | -2346.120047 | 77.85% | 39.95% | motif file (matrix) |
| 20 | 0.883 | T C G A T A C G T C A G T A C G G T C A G T C A T C A G G A C T | 1e-939 | -2163.130678 | 58.05% | 22.73% | motif file (matrix) |
| 21 | 0.853 | T C G A C T G A C T G A C G T A C T A G T C A G C T A G T C A G C G T A C G T A T A C G A G C T | 1e-904 | -2081.859586 | 47.76% | 15.55% | motif file (matrix) |
| 22 | 0.817 | C G T A A T G C G C A T C G A T A G T C G A T C G A T C G A T C G C A T G C A T G A C T | 1e-807 | -1860.040308 | 46.10% | 15.77% | motif file (matrix) |
| 23 | 0.794 | C T A G A C T G A C G T A C G T A G T C A G T C A C G T A G T C | 1e-747 | -1720.323935 | 49.27% | 19.04% | motif file (matrix) |
| 24 | 0.843 | C T G A C G T A C G T A C T A G T C G A C T A G A C T G C G T A C G T A T A C G | 1e-714 | -1644.624579 | 46.12% | 17.25% | motif file (matrix) |
| 25 | 0.835 | A T G C C G A T C G A T A T G C G T A C A C G T A T C G A C G T | 1e-639 | -1471.484027 | 22.44% | 3.99% | motif file (matrix) |
| 26 | 0.803 | G A C T G A T C C G T A A T G C C G A T C G A T T A G C A G T C G A C T G T A C G T C A G T C A | 1e-523 | -1206.176243 | 24.92% | 6.41% | motif file (matrix) |
| 27 | 0.785 | T A C G T C G A T C G A T C G A C G T A C T A G C T A G C T A G C T A G C G T A C G T A T A C G G A C T | 1e-504 | -1162.712416 | 30.51% | 9.96% | motif file (matrix) |
| 28 | 0.800 | A G T C A G T C C G A T A C G T A G T C A G T C A C T G A C T G | 1e-434 | -1001.285226 | 19.23% | 4.44% | motif file (matrix) |
| 29 | 0.784 | A T G C G A C T A G C T A G T C A G T C A G C T A G T C G A C T G A C T | 1e-380 | -875.247030 | 21.41% | 6.30% | motif file (matrix) |
| 30 | 0.781 | T C G A T C A G A G T C T C A G A T C G C G T A C G T A | 1e-376 | -866.646140 | 37.28% | 17.10% | motif file (matrix) |
| 31 | 0.783 | A T C G A G C T G C A T A C G T T A G C A G T C A C T G C T A G T A G C A C G T | 1e-297 | -685.094645 | 20.08% | 6.77% | motif file (matrix) |
| 32 | 0.839 | C T G A A C T G C G A T A G C T A G T C A G T C A G T C | 1e-270 | -623.234931 | 31.81% | 15.47% | motif file (matrix) |
| 33 | 0.768 | G A T C C T G A A T G C G T C A A C G T A T G C G A T C C A T G A T C G A C G T A G C T | 1e-155 | -358.352494 | 7.25% | 1.66% | motif file (matrix) |
| 34 | 0.785 | G A C T A G C T C G A T C G A T T A G C A G T C A C T G A C T G G A C T | 1e-136 | -313.479529 | 9.46% | 3.12% | motif file (matrix) |
| 35 | 0.797 | C T A G T A G C G T A C A C T G C T A G C T G A C G T A T C G A C T A G T G A C T C A G | 1e-128 | -296.537253 | 6.87% | 1.80% | motif file (matrix) |
| 36 | 0.754 | T C G A C G T A C G A T C T G A T A G C T C A G A C T G G C T A C G T A | 1e-89 | -205.712524 | 5.11% | 1.42% | motif file (matrix) |
| 37 | 0.798 | A C T G C T G A A C T G A C G T A C G T A G T C A G T C A C G T A C T G A C G T C A G T G A C T | 1e-58 | -134.826045 | 2.89% | 0.70% | motif file (matrix) |
